# Editorial: Signaling Pathways in Embryonic Development

**DOI:** 10.3389/fcell.2017.00076

**Published:** 2017-08-31

**Authors:** Juan J. Sanz-Ezquerro, Andrea E. Münsterberg, Sigmar Stricker

**Affiliations:** ^1^Department of Molecular and Cellular Biology, National Center for Biotechnology (CSIC) Madrid, Spain; ^2^School of Biological Sciences, University of East Anglia Norwich, United Kingdom; ^3^Institute for Chemistry and Biochemistry, Freie Universität Berlin Berlin, Germany

**Keywords:** signaling pathways, embryo development, mechanotransduction, notch signaling pathway, Wnt Signaling Pathway, Shh signaling

The formation of a complex multicellular organism from a single cell is one of the most amazing processes of biology. Embryonic development is characterized by the careful regulation of cellular behaviors such that cells proliferate, migrate, differentiate, and form tissues at the correct place and time. These processes are genetically controlled and depend both on the history of cells, their lineage, and on the activities of signaling pathways, which coordinate the cell interactions leading to organogenesis.

A limited number of key signaling pathways—Fgf, Hedgehog, Wnt, TGFß, Notch among the most important—operate during development, acting repeatedly at different times and in different regions in the embryo and eliciting diverse cellular responses. This raises the question of how cells integrate all the information they receive and can respond in cell type-specific ways to the same signals. Classical concepts in embryology such as organizers (groups of cells producing instructive signals) and competence (ability of cells to respond) can now be analyzed in molecular terms. In recent years many advances have been made in identifying the signals acting during embryo development and understanding their properties and functions, which is equally of relevance for human pathology and evolution. An important discovery is the conservation of signals and mechanisms, not only in evolutionary terms (similar genes and signals acting in distant organisms), but also in the repeated use of the same signaling pathways at different times and places in the embryos. Moreover, many of those mechanisms are involved in adult tissue homeostasis and regeneration.

Understanding developmental signaling pathways is important for several reasons. It gives us information about basic mechanisms of cell function and interactions needed for morphogenesis and organogenesis. It uncovers the basis of congenital malformations, since errors at any step of cell signaling during development are a major cause of defects. Fundamental insight also gives us clues to understand the mechanisms operating in evolution that explain diversity in form and function. And finally, it allows the identification of possible causes of disease in the adult organism (such as cancer or degenerative diseases) pinpointing possible targets for therapeutic approaches.

In this context, the aim of the Frontiers research topic “Signaling pathways in embryonic development” has been to provide a forum for experts in cell and developmental biology to share recent advances in the field of signaling during embryonic development. Sixteen articles in a variety of formats are united in this Topic, offering a valuable collection for researchers looking for an update in the knowledge of signaling pathways operating during embryogenesis. The works, focused mainly on vertebrates, explore different aspects of this theme from cell communication to organ formation and have implications for areas as distant as evolution or pathology.

Among the signaling pathways with important and widespread roles in development is the Wnt pathway, comprising a family of ligands with homology to wingless in Drosophila. Wnts can bind to multiple receptor complexes and trigger several downstream signaling cascades [including the so-called canonical WNT/β-catenin dependent signaling pathway, the non-canonical WNT/planar cell polarity (PCP), and the WNT/Ca^2+^ pathways], illustrating how the same signal can elicit diverse cellular responses depending on the cell type, context, and developmental timing. Fujimura reviews the role of canonical Wnt signaling in eye development, highlighting the important roles it plays in patterning of ocular tissue, differentiation of retinal pigment epithelium, and morphogenesis of the optic cup. Importantly, mis-regulation of the signaling cascade can lead to eye malformations and disease. Gentzel and Schambony review a group of core intracellular effectors of the Wnt pathway, disheveled (DVL) proteins, which comprise three members in vertebrates. Although all DVLs share a common basic function in Wnt signaling, the expression patterns, and functions of the different isoforms are not totally redundant and have also diverged between different species, suggesting they play specific roles depending on the tissue distribution and specific interactions. Again, mutations in DVL genes can cause human congenital disease, highlighting their important role in development. Additionally, Berger et al. review the role of PTK7 (protein tyrosine kinase 7, a transmembrane receptor) in the fine-tuning of the Wnt signaling network. Its functions in establishing cell polarity, regulation of cell movements, and migration are also essential for development and disease, particularly in cancer and metastasis.

Another important signaling pathway is Notch, a transmembrane protein that mediates juxtacrine cell-cell communication. Notch has many functions in organ formation and adult homeostasis, including cell determination and stem cell maintenance. Carrieri and Dale review the particularly well-studied function of Notch in somitogenesis and also present recent data on the role of FBXW7 protein in regulating the turnover of Notch intracellular domain (NICD, the effector of the pathway), in development and cancer. This relates to an often-overlooked essential point in signaling, which is the termination of activation and resetting of the components, allowing the cells to become competent again. Multiple mechanisms of regulation exist (positive and negative feedback loops) that allow a fine control of signaling pathways at different steps of the intracellular cascades.

Crosstalk between the limited numbers of signaling pathways is a mechanism that allows cells to respond differently to the same signal, producing the diverse cellular behaviors that are needed to build tissues and organs. A new example of this is provided by Bernatik et al. reporting on the role of the BMP antagonist Noggin in sensitizing cells and potentiating the activation of non-canonical Wnt signaling in skeletal development. They also provide evidence for a genetic interaction between these two pathways, which are involved in human congenital malformations.

The role of specific signaling pathways in the formation of particular organs is discussed in other articles. Díez del Corral and Morales review the multiple roles of Fgf signaling in the developing spinal cord. This important structure of the nervous system arises from neural derivatives of an early neuromesodermal population located at the caudal part of the embryo. Extension of this region is coupled to spinal cord formation and several essential processes such as neurogenesis, ventral patterning or neural crest specification are controlled by Fgf signaling. These embryonic functions of Fgfs could be related to its ability to promote regeneration in the injured spinal cord of adults.

Signalling pathways often converge on controlling the expression of transcription factors, which regulate cell fate specification. The integration of Notch signaling and bHLH transcription factors during inner ear development is analyzed by Gálvez et al. which also highlight that these same mechanisms are involved in hair cell regeneration, opening avenues for possible therapeutic approaches in hearing impairment. Ear development is also the topic reviewed by Magariños et al. They present evidence for a crucial role of autophagy, the regulated process of degradation, and recycling of cellular components, in vertebrate inner ear formation.

The limb is a classic model in embryology and some of the most important discoveries related to the roles of signaling pathways in pattern formation, growth, and differentiation have been made studying limb development. Tickle and Towers review the role of Shh in this process, a paradigm of how signals control and integrate tissue pattern and growth. They also discuss the implications of this important pathway for congenital malformations in humans and for the generation of limb morphological diversity during evolution. Montero et al. also treat this evolutionary aspect in their perspective article. They present a detailed analysis of Sox9 expression in developing digits of several species. This transcription factor, regulated by signaling pathways such as BMPs, Tgfßs, or Fgfs is involved in formation of the chondrogenic template of the skeleton. Differences in Sox9 expression patterns among species that have specific morphologies may reflect differences in signaling pathways controlling its expression. Also related to skeletal development, Amara et al. show that the effects of Calcium/Calmodulin dependent kinase II (CAMKII), an effector for Ca+2 -dependent signal transduction, in promoting chondrogenic differentiation seems to be specific for chicken embryos. This function is not observed in the mouse, thus highlighting the existence of differences in signaling functions and regulation among different species.

Integration of extrinsic and intrinsic regulatory cues is essential for organ formation. Dueñas et al. review the role of signals, transcription factors and cellular processes in the formation of the epicardium. This is the external-most layer of the heart that serves not only as the outer cover for this organ, but also seems to play a role in regeneration. Thus, understanding the basis of its development may have important therapeutic implications. Two articles deal with muscle development. Hernandez-Torres et al. review the role of Pitx2 in embryonic and adult myogenesis. A hierarchy of transcription factors controls skeletal muscle differentiation and Pitx2 plays an important role in the regulation of this process. Importantly, it also seems to be involved in the establishment and function of satellite cells, the stem cells resident in adult muscle, thus opening new avenues for development of regenerative therapies. Additionally, Nassari et al. review the role of connective tissues in muscle development. Apart from the intrinsic molecular signals mentioned above, the interaction of muscle cells with surrounding tissues (bone, cartilage, tendon, and ligament) is critical for the correct assembly of the musculoskeletal system during development and for maintaining adult homeostasis.

An emerging theme in developmental biology is the control of tissue morphogenesis by physical forces (mechanotransduction). Valdivia et al. review the mechanical control of myotendinous junction formation and tendon differentiation, highlighting again the importance of the interplay between chemical and mechanical signaling during embryogenesis. In the same line, Stricker et al. provide a timely discussion reminding us that cells in embryos and adult organisms are not present in isolation, but embedded in extracellular matrices into complex tissues. Cells attach to the ECM and sense its mechanical properties. Typically, experimental *in vitro* conditions do not fully reproduce this environment, which is however critical for the physiological cellular responses to signaling cascades. The challenge for the future is to try and integrate as many interactions as possible when designing experiments.

We hope that the articles in this topic will be of interest to researchers working in development and cell biology, fuelling discussion on this area and opening new avenues for thinking and investigation.

## Author contributions

JS was the Guest editor of this Research Topic, inviting co-editors AM and SS and working with them to define the subjects to be treated. They identified and invited leaders in specific research fields to contribute their work to the Research Topic. They acted as handling editors of manuscripts in the topic. JS wrote the Editorial with input from the other co-editors.

### Conflict of interest statement

The authors declare that the research was conducted in the absence of any commercial or financial relationships that could be construed as a potential conflict of interest.

